# Navigating the Challenges of Community Supports Services: A Focus Group for Black Family Caregivers

**DOI:** 10.1002/alz.090384

**Published:** 2025-01-09

**Authors:** Florence U Johnson

**Affiliations:** ^1^ University of Michigan, Ann Arbor, MI USA

## Abstract

This qualitative study highlights the challenges faced by Black family caregivers who provide personal care to dementia patients without remuneration or training. Caring for a person with dementia can be stressful and negatively impact the caregiver’s health. Black family caregivers are particularly vulnerable to stress‐related mental health issues. Community support services (CSS) such as support groups, help‐seeking, respite, and dementia training can help address these issues. CSS can also help delay or avoid institutionalization and keep the person living with dementia and their community‐dwelling caregivers safe in their homes.

The study used the RaDAR method to analyze and develop themes from the focus group interviews of six participants. The focus group participants validated the hypothesis that Black caregivers are less likely to use CSS, particularly respite and support groups. Financial constraints and lack of free time were reported as the main barriers to support groups and respite use.

The study concludes that CSS can help address the mental health issues of Black family caregivers. However, financial constraints and lack of free time are significant barriers to CSS use. The study recommends that CSS providers consider offering flexible and affordable services that cater to the needs of Black family caregivers. The study also recommends that policymakers consider providing financial support to CSS providers to enable them to offer affordable services to Black family caregivers.

